# Differential Modulation of JAK/STAT3 Signaling and BCL-2 Family Proteins by Tetracycline Analogues in Leukemia Models

**DOI:** 10.3390/pharmaceutics18040415

**Published:** 2026-03-28

**Authors:** Zienab M. Hassan, Doste R. Mamand, Hoda W. El-Gawly, Nagla A. El-Sherbeeny, Hala M. F. Mohammad, Mohamed K. Elkherbetawy, Oscar P. B. Wiklander, Moustapha Hassan

**Affiliations:** 1 Department of Clinical Pharmacology, Faculty of Medicine, Suez Canal University, Ismailia 41522, Egypt; 2 Experimental Cancer Medicine, Division of Biomolecular and Cellular Medicine (BCM), Department of Laboratory Medicine, Karolinska Institutet, 141 86 Huddinge, Sweden; 3 Department of Laboratory Medicine, Division of Biomolecular and Cellular Medicine, Karolinska Institutet, 171 77 Stockholm, Sweden; 4 Breast Center, Karolinska Comprehensive Cancer Center, Karolinska University Hospital, 171 76 Stockholm, Sweden; 5 Biology Research Center, Research Center, University of Zakho, Zakho 42002, Duhok, Kurdistan Region, Iraq; 6 Department of Pathology, Faculty of Medicine, Suez Canal University, Ismailia 41522, Egypt

**Keywords:** JAK2/STAT3, tetracycline-3 (COL-3), doxycycline (DOX) and minocycline (MIN), leukemia

## Abstract

**Background/Objectives**: Drug repurposing offers a time- and cost-efficient strategy for accelerating the development of anticancer therapies by leveraging the established safety profiles of existing pharmaceuticals. This study aimed to investigate the anticancer potential of three tetracycline analogues chemically modified tetracycline-3 (COL-3), doxycycline (DOX), and minocycline (MIN) in leukemia models, with a particular focus on their cytotoxic effects and modulation of the JAK2/STAT3 signaling pathway. **Methods**: Cytotoxicity was evaluated in K562, KG-1a and Jurkat cell lines using luminescence-based viability assays, whereas the mechanisms of cell death were analyzed by Annexin-V/7-AAD staining and Western blotting. **Results**: COL-3 displayed the highest cytotoxic potency across all cell lines, with Jurkat cells showing the greatest overall sensitivity. Flow cytometry revealed that tetracycline analogues primarily induced apoptosis, although the molecular mechanisms differed between cell lines. In K562 and KG-1a cells, apoptosis occurred largely through JAK2/STAT3-independent mechanisms, involving differential regulation of BCL-2 family proteins: COL-3 reduced BCL-2 expression, whereas DOX and MIN increased BAX expression. In contrast, Jurkat cell apoptosis correlated with suppression of phosphorylated JAK2 and STAT3 and downregulation of BCL-2, implicating a JAK2/STAT3-dependent mechanism. **Conclusions**: Taken together, these findings demonstrate that tetracycline analogues exert cell line-specific anticancer activities through distinct molecular pathways. Among them, COL-3 emerges as the most potent analogue and acts through both JAK/STAT-dependent and -independent mechanisms. This work supports further investigation of COL-3 as a candidate for drug repurposing strategies in hematological malignancies.

## 1. Introduction

Drug repurposing, also known as drug repositioning, has emerged as an attractive strategy for accelerating the development of cancer therapeutics. By identifying new indications for approved drugs with known safety, pharmacokinetic and toxicological profiles, this approach reduces the time and cost associated with traditional drug discovery pipelines [1,2,3]. Increasing evidence supports the feasibility of repurposing antimicrobials as antineoplastic agents, particularly in settings where resistance, toxicity and limited durable responses remain significant clinical challenges [4,5,6].

Leukemia encompasses a heterogeneous group of hematological malignancies characterized by uncontrolled proliferation of hematopoietic cells. Although considered relatively uncommon, leukemia represents a substantial clinical burden. In the United States, approximately 61,090 new cases were reported in 2021, accounting for ~3% of all newly diagnosed cancers [7,8]. In children, leukemia constitutes nearly one-third of all cancers, with acute lymphoblastic leukemia (ALL) representing 75–80% of pediatric cases [9,10,11]. Among adults, chronic myeloid leukemia (CML) comprises ~15% of leukemia diagnoses and predominantly affects older individuals, with a median age at diagnosis of 65 years [12,13,14]. Despite advances in targeted therapy and hematopoietic stem cell transplantation, resistance, relapse and treatment-related toxicities remain major obstacles, underscoring the need for novel therapeutic strategies [15,16,17].

Recent developments in molecular oncology have deepened the understanding of signalling networks driving leukemic cell survival and proliferation. Among these, the Janus kinase/signal transducer and activator of transcription (JAK/STAT) pathway plays a pivotal role in cytokine-mediated regulation of hematopoietic cell growth, differentiation and apoptosis. Aberrant JAK/STAT activation, whether driven by mutations, cytokine microenvironments or upstream kinase dysregulation, has been widely documented across leukemia subtypes and linked to therapeutic resistance and disease progression [18,19,20]. Inhibition of JAK kinases or blockade of STAT3 activation therefore represents a rational therapeutic approach, and several small-molecule inhibitors targeting this axis are currently under clinical evaluation.

Parallel to these efforts, the tetracycline class of antibiotics—including doxycycline (DOX), minocycline (MIN) and chemically modified tetracycline-3 (COL-3) has attracted attention due to reported anti-metastatic, antiproliferative and pro-apoptotic activities in solid and hematologic malignancies [18,21,22,23,24]. Although originally developed as antimicrobial drugs, tetracyclines have been repurposed because they exhibit a broad range of biological effects beyond antibacterial activity. Previous studies have shown that DOX, MIN, and COL-3 can inhibit cancer cell proliferation, induce apoptosis, and suppress tumour migration through multiple mechanisms, including inhibition of matrix metalloproteinases (MMP-2 and MMP-9), interference with oncogenic signalling pathways, mitochondrial dysfunction, oxidative stress induction, and disruption of mitochondrial protein translation [25,26,27]. In addition, their interactions with apoptosis-regulating proteins, such as members of the BCL-2 family, suggest mechanistic links to intrinsic cell death pathways. Evidence from leukemia models further indicates that DOX can reduce leukemia cell migration and proliferation, highlighting the potential of tetracyclines as promising drug-repurposing candidates for leukemia therapy [28].

However, the extent to which tetracycline analogues modulate oncogenic signalling pathways such as JAK2/STAT3 in leukemia remains insufficiently characterized. Given the pathway’s centrality in leukemogenesis and treatment resistance, elucidating these interactions may provide insight into repurposing opportunities and reveal mechanistic dependencies that vary across leukemia subtypes.

Therefore, a significant gap in current knowledge lies in understanding whether tetracycline derivatives can directly modulate the JAK2/STAT3 signalling pathway and influence the balance between pro-apoptotic and anti-apoptotic proteins, such as BAX and BCL-2, in leukemia cells. Addressing this gap is important because dysregulation of these pathways contributes to leukemia cell survival and therapeutic resistance.

This study investigated the effects of chemically modified tetracycline-3 (COL-3), doxycycline (DOX), and minocycline (MIN) on leukemia cell lines K562, KG-1a, and Jurkat, with a particular focus on the JAK2/STAT3–BCL-2 regulatory axis. The half-maximal inhibitory concentration (IC50) of each compound was determined in all three cell lines. The mechanism of cell death was analyzed by flow cytometry to distinguish apoptotic from necrotic populations, while Western blotting was used to evaluate the effects of these compounds on JAK2/STAT3 signalling and on the expression of the apoptosis-related proteins BAX and BCL-2. Our findings demonstrate cell line-specific cytotoxic and mechanistic responses, with COL-3 showing the greatest potency among the tested analogues. Notably, COL-3 appeared to act through both JAK/STAT-dependent and independent pathways. Overall, these results improve our understanding of the anti-leukemic activity of tetracycline derivatives and support their further investigation as repurposed therapeutic agents for hematologic malignancies.

## 2. Materials and Methods

### 2.1. Materials

Doxycycline hyclate (DOX) and minocycline hydrochloride (MIN) were purchased from Sigma Aldrich (Stockholm, Sweden) as crystalline powders and dissolved in distilled water. Chemically modified tetracycline-3 (COL-3) was provided by CollaGenex Pharmaceuticals Inc. (Newtown, PA, USA) and Galderma R&D (Sophia-Antipolis, France) and dissolved in dimethyl sulfoxide (DMSO). RPMI 1640 medium supplemented with GlutaMAX™ and HEPES, fetal bovine serum (FBS), penicillin–streptomycin, phosphate-buffered saline (PBS) and other cell culture reagents were obtained from Gibco (Thermo Fisher Scientific, Waltham, XA, USA). Additional reagents included MycoAlert™ Mycoplasma Detection Kit (Lonza, Basel, Switzerland), CellTiter-Glo^®^ Luminescent Cell Viability Assay (Promega, Madison, WI, USA), PE Annexin V Apoptosis Detection Kit I (BD Biosciences, Milpitas, CA, USA), and standard Western blotting materials. Antibodies against JAK2, P-JAK2, STAT3, P-STAT3, BCL-2, BAX and β-actin and corresponding HRP-conjugated secondary antibodies were sourced according to manufacturers’ specifications. Catalogue numbers for all reagents are provided in the Supplementary Materials.

### 2.2. Cell Culture

The leukemia cell lines K562 (ATCC, CCL-243), KG-1a (ATCC, CCL-246.1), and Jurkat (ATCC, TIB-152) were selected to represent major hematological malignancy subtypes across both myeloid and lymphoid lineages. K562 cells model Chronic Myeloid Leukemia (CML), KG-1a cells represent Acute Myeloid Leukemia (AML) with a primitive progenitor phenotype, and Jurkat cells serve as a model for T-cell Acute Lymphoblastic Leukemia (T-ALL). Together, these lines provide a representative panel of myeloid (CML/AML) and lymphoid (T-ALL) leukemias, reflecting key aspects of leukemia heterogeneity and enabling evaluation of therapeutic effects and signalling pathway modulation across distinct leukemia subtypes. K562, KG-1a and Jurkat cell lines were maintained in RPMI 1640 medium supplemented with 10% FBS and 1% penicillin–streptomycin at 37 °C in a humidified incubator with 5% CO_2_. KG-1a cells were cultured with 20% FBS. Cells were used at passage numbers below 10 to ensure consistency and were routinely screened for mycoplasma contamination using MycoAlert™, with only mycoplasma-free cultures included in experiments.

### 2.3. Cell Starvation and Activation of Jurkat Cells

Jurkat cells were serum-starved for 4 h in RPMI 1640 to facilitate subsequent activation of the JAK2/STAT3 pathway. Activation was induced using pervanadate (PERV), a protein tyrosine phosphatase inhibitor that enhances tyrosine phosphorylation and thereby promotes JAK2 and STAT3 activation. After starvation, cells were treated with an activation mixture containing PERV and FBS for 15 min at room temperature, washed with PBS and resuspended in complete medium prior to treatment with tetracycline analogues.

### 2.4. Drug Treatment Conditions

Cells were divided into eight experimental groups per cell line, consisting of untreated controls, vehicle controls (0.1% DMSO), and two concentrations each of COL-3, DOX and MIN. Concentration ranges were selected based on pilot experiments to approximate IC_50_ values. Jurkat cells were additionally analyzed in activated and non-activated states. Detailed concentration schemes for each cell line are provided in Supplementary Tables S1–S3.

### 2.5. Cell Viability Assay

Cytotoxicity was assessed using the CellTiter-Glo^®^ Luminescent Cell Viability Assay according to the manufacturer’s instructions. Briefly, 1.5 × 10^4^ cells were seeded per well in opaque 96-well plates and treated with drug concentrations for 24 h. CellTiter-Glo^®^ reagent (Promega, Madison, WI, USA) was added at a 1:1 ratio, mixed for 2 min and incubated for 10 min at room temperature to stabilize luminescence. Luminescence was recorded using a SpectraMax i3X plate reader (Molecular Devices LLC, San Jose, CA, USA) and analyzed with SoftMax Pro 7. IC_50_ values were determined from nonlinear regression fits.

### 2.6. Assessment of Cell Death Mechanisms

Apoptosis and necrosis were evaluated by Annexin V/7-AAD staining. After treatment for 4 h and 24 h, cells were washed twice with cold PBS, resuspended in binding buffer and stained with PE Annexin V and 7-AAD for 15 min at room temperature in the dark. Samples were analyzed within 1 h using a MACSQuant Analyzer 10 (Miltenyi Biotec, Bergisch Gladbach, Germany). Populations were gated as viable (Annexin V^−^/7-AAD^−^), early apoptotic (Annexin V^+^/7-AAD^−^), late apoptotic (Annexin V^+^/7-AAD^+^) or necrotic (Annexin V^−^/7-AAD^+^). Compensation controls were included for all fluorochromes.

### 2.7. Protein Extraction and Western Blotting

Cells (1 × 10^7^) were lysed in RIPA buffer supplemented with protease and phosphatase inhibitors and incubated on ice for 30 min. Protein concentrations were determined using a BCA assay. Equal amounts of protein were mixed with sample buffer containing β-mercaptoethanol, denatured at 95 °C for 5 min and separated on 4–15% SDS-PAGE gels. Proteins were transferred onto PVDF membranes overnight at 4 °C, blocked with 5% BSA in TBS-T for 2 h and probed with primary antibodies against JAK2, phosphorylated JAK2 (P-JAK2), STAT3, phosphorylated STAT3 (P-STAT3), BCL-2, BAX and β-actin. Membranes were incubated with HRP-conjugated secondary antibodies, washed and visualized using a LI-COR scanner (LI-COR Biosciences, Lincoln, NE, USA). Band intensities were quantified with Odyssey or ImageJ software (v6.1).

### 2.8. Statistical Analysis

Data are presented as mean ± standard deviation (SD) unless otherwise indicated. Comparisons among multiple groups were performed using one-way ANOVA followed by post hoc tests (Dunnett’s multiple comparisons test was used to determine the statistical significance of differences between the untreated control (or DMSO control) and the various drug-treated groups). Differences were considered statistically significant at *p* < 0.05. IC_50_ values were calculated using nonlinear curve fitting. All analyses were performed using GraphPad Prism 10.

## 3. Results

### 3.1. Cytotoxic Effects of Tetracycline Analogues in Leukemia Cell Lines

The cytotoxicities of COL-3, DOX and MIN were evaluated in K562, KG-1a and Jurkat cells using CellTiter-Glo™. All compounds reduced cell viability in a dose-dependent manner, with potency varying among both analogues and cell lines.

**Figure 1:**
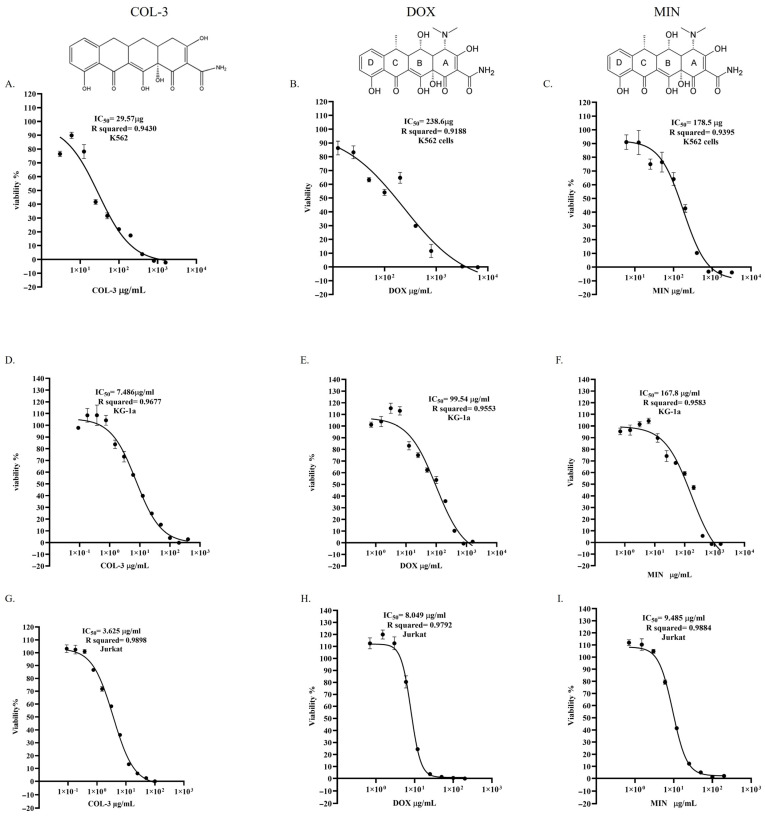
Dose-dependent effects and IC_50_ determination of COL-3, DOX, and MIN in K562, KG-1a, and Jurkat cell lines: (**A**–**C**) Dose–response curves of COL-3, DOX, and MIN in K562 cells. (**D**–**F**) Dose–response curves of COL-3, DOX, and MIN in KG-1a cells. (**G**–**I**) Dose–response curves of COL-3, DOX, and MIN in Jurkat cells. Data are presented as mean ± standard error (SE) from three independent experiments. IC_50_ values are calculated using the profile likelihood method and are reported with 95% confidence intervals (CI).

In K562 cells, COL-3 exhibited the lowest IC_50_ value (7.96 × 10^−5^ mol/L; 95% CI: 5.77 × 10^−5^–1.11 × 10^−4^; R^2^ = 0.9430), followed by MIN (3.61 × 10^−4^ mol/L; 95% CI: 2.72–4.76 × 10^−4^; R^2^ = 0.9395) and DOX (4.65 × 10^−4^ mol/L; 95% CI: 2.66 × 10^−4^–1.16 × 10^−3^; R^2^ = 0.9188) (Figure 1A–C; Supplementary Table S1). In KG-1a cells, COL-3 again showed the greatest potency (IC_50_ = 2.02 × 10^−5^ mol/L; 95% CI: 1.59–2.59 × 10^−5^; R^2^ = 0.9677), whereas DOX and MIN exhibited higher IC_50_ values of 1.94 × 10^−4^ mol/L (95% CI: 1.38–2.98 × 10^−4^; R^2^ = 0.9553) and 3.40 × 10^−4^ mol/L (95% CI: 2.47–5.38 × 10^−4^; R^2^ = 0.9583), respectively (Figure 1D–F; Supplementary Table S2). Jurkat cells displayed the greatest overall sensitivity, with IC_50_ values of 9.76 × 10^−6^ mol/L (COL-3; 95% CI: 8.62–1.10 × 10^−6^; R^2^ = 0.9898), 1.57 × 10^−5^ mol/L (DOX; 95% CI: 1.43–1.73 × 10^−5^; R^2^ = 0.9792) and 1.92 × 10^−5^ mol/L (MIN; 95% CI: 1.76–2.09 × 10^−5^; R^2^ = 0.9884) (Figure 1G–I; Supplementary Table S3). Overall, COL-3 demonstrated the highest cytotoxic potency across all three cell lines, followed by DOX and MIN, whereas Jurkat cells exhibited the greatest sensitivity and K562 cells the greatest resistance.

### 3.2. Tetracycline Analogues Induce Apoptosis with Cell Line-Specific Features

To determine the mode of cell death, Annexin-V/7-AAD staining was performed after 4 h and 24 h of exposure to each analogue.

**Figure 2:**
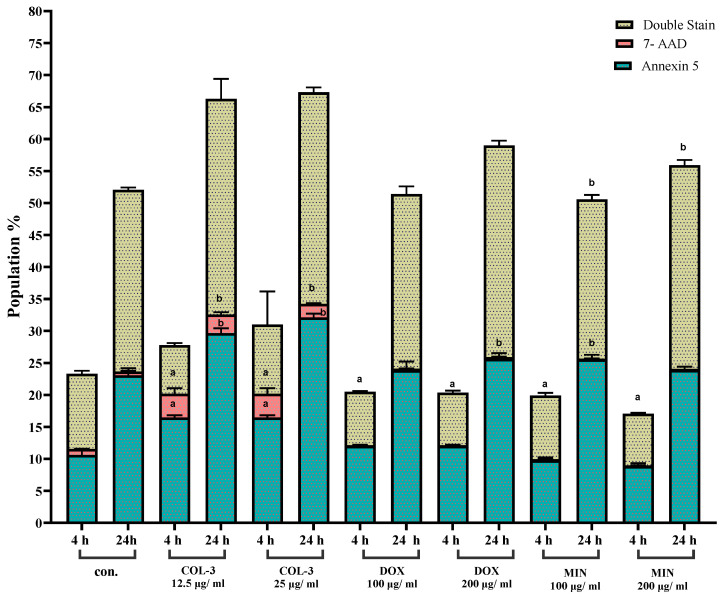
Effect of COL-3, DOX, and MIN on apoptosis in K562 cells assessed by flow cytometry. Flow cytometric analysis of K562 cells treated with COL-3 (12.5 µg/mL, 25 µg/mL), DOX (100 µg/mL, 200 µg/mL), and MIN (100 µg/mL, 200 µg/mL), showing the percentage of Annexin V-positive cells (early apoptosis), 7-AAD-positive cells (late apoptosis/necrosis), and double-stained cells (late apoptosis/necrosis). Data represent the mean ± standard error (SE) from three independent experiments. Bar graphs illustrate the percentage changes in cell populations. a: Statistically significant difference compared to 4 h control cells (*p* < 0.05). b: Statistically significant difference compared to 24 h control cells (*p* < 0.05).

In K562 cells, cells were treated with COL-3 at concentrations of 12.5 µg/mL and 25 µg/mL for 4 h and 24 h. Annexin V-positive cells showed a statistically significant increase in all treated groups compared with their respective control cells (*p* < 0.05). After 4 h of treatment with COL-3 (12.5 and 25 µg/mL), the percentage of Annexin V-positive cells increased to 16.4% compared with 10.6% in the 4 h control cells. Similarly, after 24 h of treatment with COL-3 (12.5 and 25 µg/mL), the percentage of Annexin V-positive cells increased to 29.6% and 32.1%, respectively, compared with 23% in the 24 h control cells. DOX (100 and 200 µg/mL) showed minimal changes at 4 h but resulted in a slight increase in Annexin-V^+^ cells at 24 h by 2.5% (25.6% vs. 23.1% in the control group), with late apoptotic (double-positive) populations elevated at 200 µg/mL. MIN (100 and 200 µg/mL) produced significant increases in late apoptotic cells at 24 h (e.g., 31.8% at 200 µg/mL), accompanied by reduced viability. These data indicate apoptosis as the death modality in K562 cells, with magnitude correlating with concentration and exposure time (Figure 2; Supplementary Figures S1 and S2, Supplementary Table S4).

**Figure 3:**
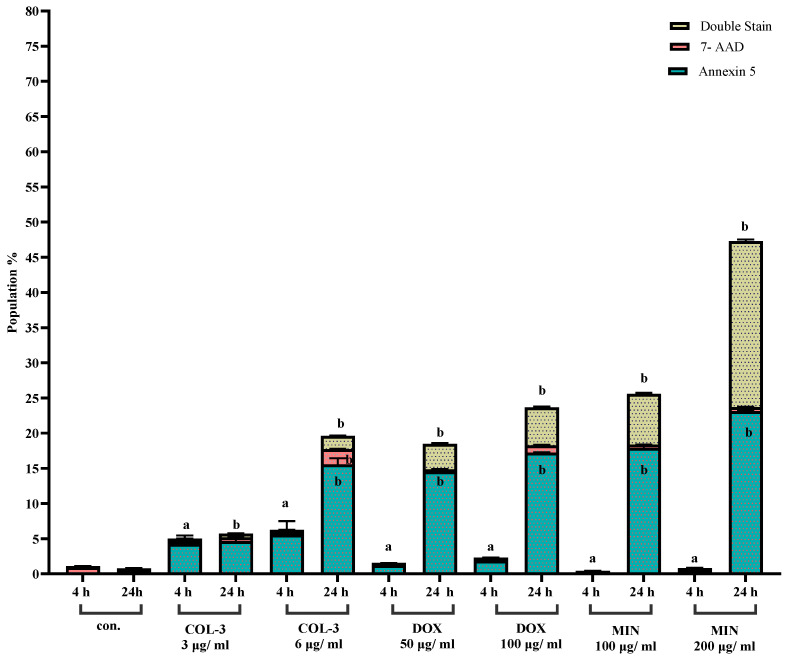
Effect of COL-3, DOX, and MIN on apoptosis in KG-1a cells assessed by flow cytometry. Flow cytometric analysis of K562 cells treated with COL-3 (3 µg/mL, 6 µg/mL), DOX (100 µg/mL, 200 µg/mL), and MIN (100 µg/mL, 200 µg/mL), showing the percentage of Annexin V-positive cells (early apoptosis), 7-AAD-positive cells (late apoptosis/necrosis), and double-stained cells (late apoptosis/necrosis). Data represent the mean ± standard error (SE) from three independent experiments. Bar graphs illustrate the percentage changes in cell populations. a: Statistically significant difference compared to 4 h control cells (*p* < 0.05). b: Statistically significant difference compared to 24 h control cells (*p* < 0.05).

Next in KG-1a Cells, COL-3 (3 and 6 µg/mL) significantly increased early apoptosis at both 4 h (4.2–5.6% vs. 0.04% control) and 24 h (4.6–15.6% vs. 0.02% control), with moderate increases in late apoptosis at 24 h. DOX (50 and 100 µg/mL) induced similar patterns, with early apoptotic populations rising from 1.2 to 1.8% at 4 h to 14.5–17.2% at 24 h. MIN (100 and 200 µg/mL) elicited substantial apoptosis at 24 h (18–23.1%) with associated increases in late apoptotic populations (7.2–23.5%). Together, these results demonstrate that all three analogues induce apoptosis in KG-1a cells in a time-dependent manner (Figure 3; Supplementary Figures S3 and S4, Supplementary Table S5).

**Figure 4:**
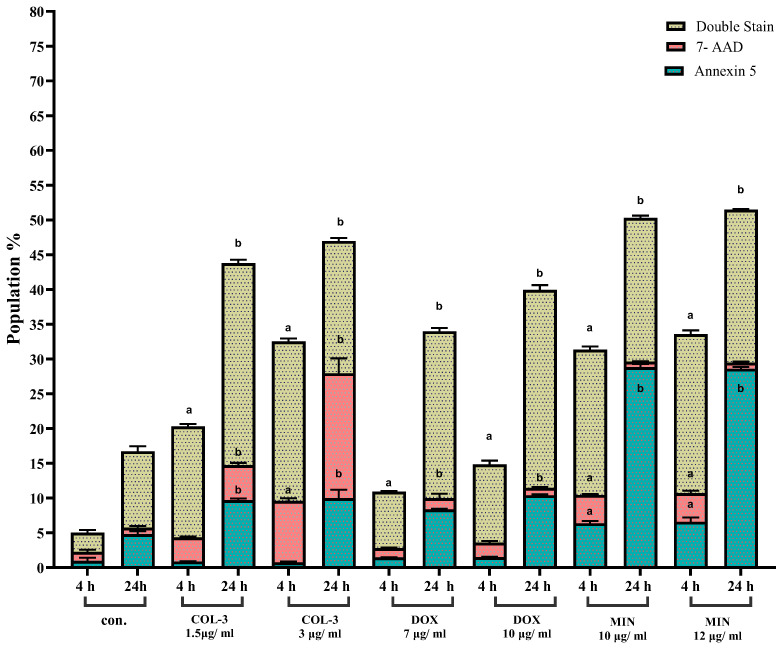
Effect of COL-3, DOX, and MIN on apoptosis in Jurkat cells assessed by flow cytometry. Flow cytometric analysis of Jurkat cells treated with COL-3 (1.5 µg/mL, 3 µg/mL), DOX (7 µg/mL, 10 µg/mL), and MIN (10 µg/mL, 12 µg/mL), showing the percentage of Annexin V-positive cells (early apoptosis), 7-AAD-positive cells (late apoptosis/necrosis), and double-stained cells (late apoptosis/necrosis). Data represent the mean ± standard error (SE) from three independent experiments. Bar graphs illustrate the percentage changes in cell populations. a: Statistically significant difference compared to 4 h control cells (*p* < 0.05). b: Statistically significant difference compared to 24 h control cells (*p* < 0.05).

In Jurkat cells, COL-3 induced marked increases in late apoptotic populations at both 4 h and 24 h (e.g., 16–29% vs. 2.5–11% control), while DOX increased double-positive populations to 24–28.5% at 24 h. MIN induced the strongest apoptotic response at early time points, with Annexin-V^+^ populations reaching 6.3–6.5% at 4 h and 29% at 24 h. These findings indicate that Jurkat cells exhibit rapid apoptotic induction in response to tetracycline analogues (Figure 4; Supplementary Figures S5 and S6, Supplementary Table S6).

## 4. Effects on the JAK2/STAT3 Pathway and BCL-2 Family Proteins

Western blotting was performed to determine whether apoptosis correlated with modulation of JAK2/STAT3 pathway components and BCL-2 family proteins.

**Figure 5:**
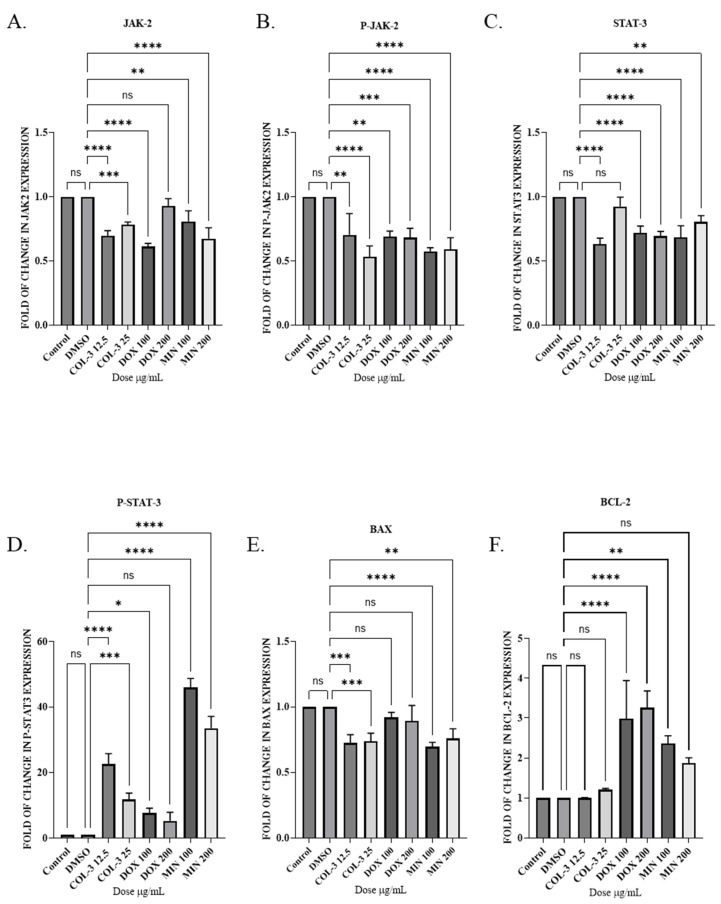
The effect of tetracycline analogues on the JAK2/STAT3 pathway in K562 cells using WB: (**A**) Expression levels of JAK2 protein following treatment with COL-3 (12.5 µg/mL, 25 µg/mL), DOX (100 µg/mL, 200 µg/mL), and MIN (100 µg/mL, 200 µg/mL). (**B**) Expression levels of P-JAK2 after treatment with COL-3, DOX, and MIN. (**C**) Expression levels of STAT3 protein in response to COL-3, DOX, and MIN. (**D**) Expression levels of P-STAT3 following treatment with COL-3, DOX, and MIN. (**E**) Expression levels of pro-apoptotic protein BAX after treatment with COL-3, DOX, and MIN. (**F**) Expression levels of anti-apoptotic protein BCL-2 in response to COL-3, DOX, and MIN. Bar graphs represent fold changes in protein expression from at least three independent experiments (mean ± SD). Statistical significance is determined using one-way ANOVA followed by Dunnett’s multiple comparison test (ns (nonsignificant), * *p* < 0.05, ** *p* < 0.01, *** *p* < 0.001, **** *p* < 0.0001).

In K562 cells, all three analogues reduced total JAK2 and P-JAK2 levels, with COL-3 and MIN exhibiting statistically significant decreases. Total STAT3 expression also declined across conditions, whereas P-STAT3 increased, reaching significance in COL-3 and MIN groups. COL-3 and MIN reduced BAX expression, while DOX and MIN increased BCL-2 levels, suggesting apoptosis occurred independently of JAK2/STAT3 suppression and was associated with BCL-2 upregulation (Figure 5; Supplementary Figure S7). The other treatment groups showed small increases in BCL-2 expressions that were not statistically significant (Figure 5F, Supplementary Figure S7).

**Figure 6:**
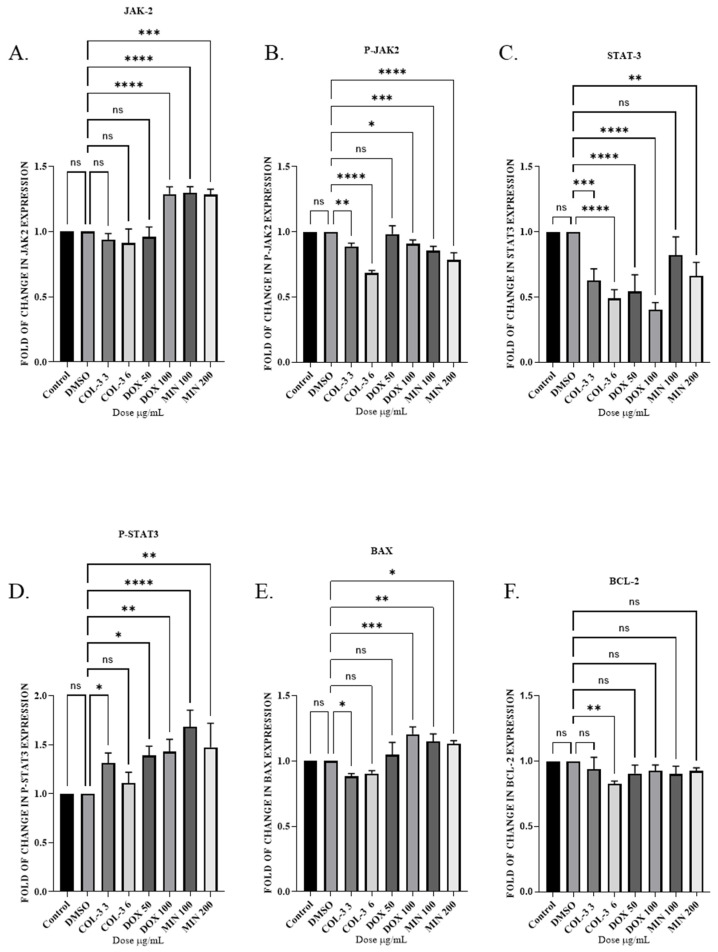
The effect of tetracycline analogues on the JAK2/STAT3 pathway in KG -1a cells using WB: (**A**) Expression levels of JAK2 protein following treatment with COL-3 (3 µg/mL, 6 µg/mL), DOX (50 µg/mL, 200 µg/mL), and MIN (100 µg/mL, 200 µg/mL). (**B**) Expression levels of P-JAK2 after treatment with COL-3, DOX, and MIN. (**C**) Expression levels of STAT3 protein in response to COL-3, DOX, and MIN. (**D**) Expression levels of P-STAT3 following treatment with COL-3, DOX, and MIN. (**E**) Expression levels of pro-apoptotic protein BAX after treatment with COL-3, DOX, and MIN. (**F**) Expression levels of anti-apoptotic protein BCL-2 in response to COL-3, DOX, and MIN. Bar graphs represent fold changes in protein expression from at least three independent experiments (mean ± SD). Statistical significance is determined using one-way ANOVA followed by Dunnett’s multiple comparison test (ns (nonsignificant), * *p* < 0.05, ** *p* < 0.01, *** *p* < 0.001, **** *p* < 0.0001).

Next in KG-1a Cells, COL-3 and DOX (50 µg/mL) modestly reduced total JAK2 expression, whereas DOX (100 µg/mL) and MIN increased it. P-JAK2 was reduced by COL-3 and MIN. STAT3 levels declined in all groups except MIN (100 µg/mL), while P-STAT3 increased significantly in DOX- and MIN-treated cells. COL-3 selectively reduced BCL-2, whereas DOX and MIN increased BAX expression, indicating apoptosis was associated with modulation of BCL-2 family proteins rather than JAK2/STAT3 inhibition (Figure 6; Supplementary Figure S8).

**Figure 7:**
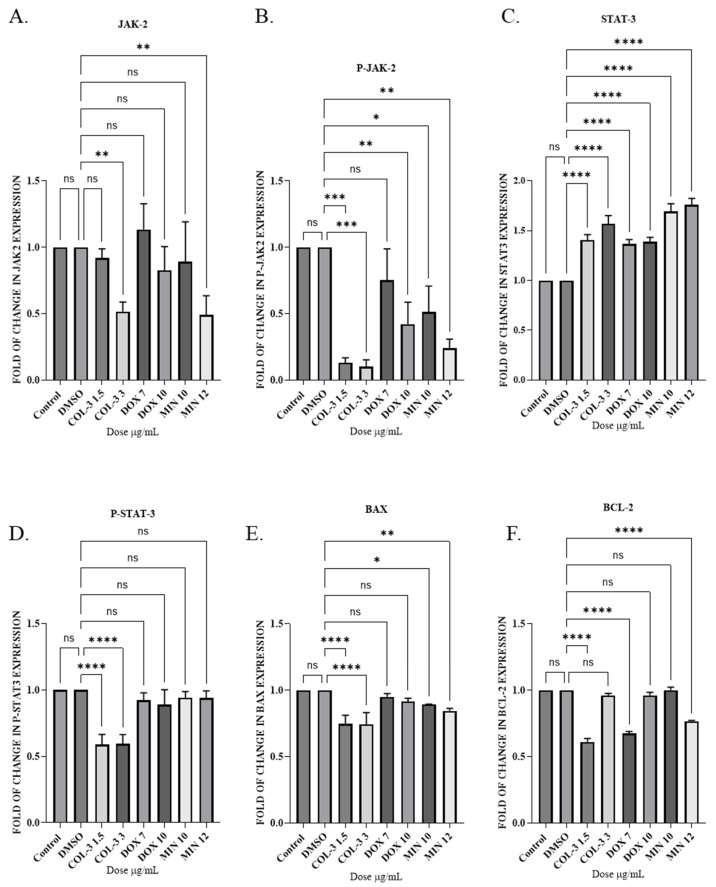
The effect of tetracycline analogues on the JAK2/STAT3 pathway in Jurkat cells using WB: (**A**) Expression levels of JAK2 protein following treatment with COL-3 (1.5 µg/mL, 3 µg/mL), DOX (7 µg/mL, 10 µg/mL), and MIN (10 µg/mL, 12 µg/mL). (**B**) Expression levels of P-JAK2 after treatment with COL-3, DOX, and MIN. (**C**) Expression levels of STAT3 protein in response to COL-3, DOX, and MIN. (**D**) Expression levels of P-STAT3 following treatment with COL-3, DOX, and MIN. (**E**) Expression levels of pro-apoptotic protein BAX after treatment with COL-3, DOX, and MIN. (**F**) Expression levels of anti-apoptotic protein BCL-2 in response to COL-3, DOX, and MIN. Bar graphs represent fold changes in protein expression from at least three independent experiments (mean ± SD). Statistical significance is determined using one-way ANOVA followed by Dunnett’s multiple comparison test (ns (nonsignificant), * *p* < 0.05, ** *p* < 0.01, *** *p* < 0.001, **** *p* < 0.0001).

Subsequently in Jurkat cells, all analogues reduced total and P-JAK2 levels, with P-JAK2 suppression reaching statistical significance in all groups except DOX (7 µg/mL). P-STAT3 levels were reduced by all treatments, with COL-3 showing significant effects. Total STAT3 levels increased across groups. BCL-2 expression was significantly reduced by COL-3, DOX and MIN (12 µg/mL), whereas BAX expression decreased following COL-3 and MIN treatment. These data indicate that apoptosis in Jurkat cells correlates with inhibition of the JAK2/STAT3 pathway and downregulation of BCL-2 (Figure 7; Supplementary Figure S9).

## 5. Discussion

This study demonstrates that the tetracycline analogues COL-3, DOX and MIN exhibit differential cytotoxic and mechanistic effects in leukemia cell lines and modulate apoptotic pathways through both JAK2/STAT3-dependent and -independent mechanisms. Among the three compounds, COL-3 consistently showed the greatest potency, while Jurkat cells displayed the highest sensitivity. These findings extend emerging evidence that tetracycline derivatives possess anticancer activities distinct from their traditional antimicrobial roles and support their consideration as candidates for drug repurposing strategies in hematologic malignancies.

Previous studies have reported variable antitumor responses to tetracyclines in both solid and hematologic cancers, including effects on mitochondrial function, transcriptional regulation and apoptosis induction [23,24,29,30,31]. The IC_50_ values observed in the present work are broadly consistent with trends described by Fares et al. (2015), although absolute values differed, likely reflecting assay platforms and detection sensitivity [32]. The use of CellTiter-Glo in this study, which quantifies ATP and is considered more sensitive for assessing metabolic activity, may account for some of these differences. Collectively, these data reinforce that tetracycline analogues exhibit anti-leukemic effects in vitro and that their potency is influenced by both drug structure and leukemia subtype.

Mechanistically, tetracycline analogues produced distinct effects on the JAK2/STAT3 axis and BCL-2 family proteins. In K562 and KG-1a cells, apoptosis did not correlate with suppression of P-JAK2 or P-STAT3, suggesting that cell death occurred largely through JAK/STAT-independent mechanisms. Instead, differential modulation of BCL-2 family proteins was observed: COL-3 reduced BCL-2 expression in KG-1a cells, whereas doxycycline (DOX) and minocycline (MIN) increased BAX levels. These findings are consistent with reports that mitochondrial dysfunction and oxidative stress contribute to tetracycline-induced cytotoxicity, with BCL-2 family proteins acting as key mediators of apoptosis [24,26,28].

Interestingly, in some cases increased apoptosis occurred alongside elevated BCL-2 expression, which may reflect several mechanisms. Compensatory feedback regulation may lead cells to upregulate survival proteins under drug-induced stress despite inhibition of upstream signalling pathways [33]. Cell population heterogeneity may also contribute, as Western blot analysis reflects average protein levels across the entire cell population, potentially masking apoptotic and resistant subpopulations [34]. Additionally, post-translational modifications such as cleavage or phosphorylation of BCL-2 family proteins can alter their functional activity, allowing apoptosis to proceed despite elevated BCL-2 levels [35]. Finally, apoptosis may involve alternative mitochondrial pathways independent of the classical BCL-2/BAX axis, mediated by other BCL-2 family members such as BAK or BAD [36].

Serum starvation followed by pervanadate (PERV) stimulation was used to activate the JAK2/STAT3 pathway in Jurkat cells. Serum starvation reduces basal signalling and synchronizes cells, whereas PERV induces rapid tyrosine phosphorylation, mimicking the constitutive kinase signalling frequently observed in T-cell acute lymphoblastic leukemia (T-ALL) and other hematological malignancies. Using this activated model, Jurkat cells demonstrated a clear association between increased apoptosis and suppression of P-JAK2 and P-STAT3, accompanied by reduced BCL-2 expression. Given that constitutive STAT3 activation is strongly linked to survival signalling in T-cell malignancies, the inhibition of this pathway suggests that COL-3, DOX, and MIN exert JAK/STAT-dependent anti-leukemic effects in lymphoid contexts. Notably, these findings differ from those observed in non-activated Jurkat cells [29], indicating that cellular activation state and pathway priming significantly influence drug response. Collectively, these results support the concept that tetracycline analogues can induce apoptosis through signalling-dependent mechanisms that vary according to leukemia subtype and pathway activity, highlighting the importance of JAK/STAT signalling status in determining therapeutic sensitivity.

From a translational perspective, the heterogeneous responses observed across leukemia models highlight both opportunities and challenges for repurposing tetracycline analogues. The superior potency of COL-3, coupled with its dual pathway engagement, suggests that it may hold particular promise for further evaluation. Although DOX and MIN were less potent and required higher concentrations to elicit effects, their established clinical use, favourable safety profiles and widespread availability make them appealing candidates for combination or adjunctive approaches. Notably, prior work has demonstrated synergistic interactions between tetracyclines and cytotoxic agents in solid tumour models [29], indicating that combinatorial strategies in leukemia warrant investigation.

This study has several limitations. First, all experiments were conducted in established cell lines, which may not fully recapitulate primary leukemia biology. Second, no in vivo validation was performed, and pharmacokinetic considerations such as achievable plasma concentrations may influence translational feasibility. Third, this study did not interrogate downstream transcriptional consequences of STAT3 inhibition or mitochondrial function, which could provide additional mechanistic insight. In addition, some of the in vitro concentrations of doxycycline and minocycline used in this study (100–200 µg/mL) exceed clinically achievable plasma levels and therefore represent supra-therapeutic conditions. Pharmacokinetic studies indicate that standard oral doses of doxycycline typically produce peak plasma concentrations of approximately 2–5 µg/mL in humans [37,38]. In contrast, the COL-3 concentrations tested (1.5 to 6 µg/mL) are closer to reported clinical plasma levels [39]. Therefore, caution should be exercised when extrapolating these in vitro findings to clinical settings. Addressing these limitations through vivo models, primary patient samples, and pathway-level profiling will be important to better define the therapeutic relevance of these findings.

Future work should prioritize evaluating COL-3 in preclinical leukemia models to determine dosing, tolerability and functional pathway engagement in vivo. Investigation into combinatorial regimens, particularly with JAK/STAT inhibitors or BCL-2 family modulators such as venetoclax, may yield synergistic effects given the mechanistic intersections identified. Furthermore, profiling leukemia subtypes with defined JAK/STAT activation states may enable stratification strategies for precision repurposing.

In conclusion, this study provides evidence that tetracycline analogues exert anti-leukemic effects through cell line-specific mechanisms and identifies COL-3 as the most potent candidate for further investigation. While additional validation is required, these findings support the continued exploration of tetracycline derivatives as repurposed agents in hematologic malignancies and highlight the value of targeting survival signalling pathways such as JAK/STAT3 in leukemia.

## 6. Conclusions

This study demonstrates that tetracycline analogues exert anti-leukemic effects through distinct molecular mechanisms that vary across leukemia cell lines. COL-3 showed the highest cytotoxic potency and engaged both JAK/STAT-dependent and -independent apoptotic pathways, whereas DOX and MIN induced apoptosis primarily through modulation of BCL-2 family proteins. Jurkat cells exhibited apoptosis associated with suppression of P-JAK2 and P-STAT3 and downregulation of BCL-2, suggesting pathway dependence in lymphoid cells. In contrast, apoptosis in K562 and KG-1a cells occurred independently of JAK/STAT signalling and involved differential regulation of BCL-2 or BAX. These findings highlight mechanistic heterogeneity in leukemia cell responses to tetracycline analogues and identify COL-3 as the most promising candidate for further exploration. While additional validation in primary models and in vivo systems is required, this work supports continued evaluation of tetracycline derivatives as repurposed candidates in hematological malignancies.

## Data Availability

The original contributions presented in this study are included in the article/Supplementary Materials. Further inquiries can be directed to the corresponding authors.

## Supplementary Materials

The following supporting information can be downloaded at: https://www.mdpi.com/article/10.3390/pharmaceutics18040415/s1.

## Abbreviations

The following abbreviations are used in this manuscript:

COL-3	tetracycline-3
DOX	doxycycline
MIN	minocycline
