# Supplementary material for: Differential Modulation of JAK/STAT3 Signaling and BCL-2 Family Proteins by Tetracycline Analogues in Leukemia Models
Source: Pharmaceutics. 2026 Mar 28;18(4):415. doi: 10.3390/pharmaceutics18040415 (PMC13118528; doi:10.3390/pharmaceutics18040415)
Supplement: Supplementary file 1 [file pharmaceutics-18-00415-s001.zip › pharmaceutics-4177875-supplementary.pdf]

Table S1. Different amounts of chemically modified tetracycline-3 (COL-3), Doxycycline (DOX), and minocycline (MIN) affect the health of K562 cells. The data are shown as the mean

The used COL-3 dose μg/ml	The Percentage of viable cells after COL-3 treatment (Mean ±SD)	The used DOX and MIN doses μg/ml	The Percentage of viable cells after DOX treatment (Mean ±SD)	The Percentage of viable cells after MIN treatment (Mean ±SD)
0	100 ±16.88	0	100 ± 16.87	100 ± 16.87
3	76.44 ± 3.92	12.5	86.28 ± 10	90.75 ± 17.55
6	89.84 ± 4.577	25	83.24 ± 9.38	74.97 ± 7.47
12.5	78.18 ± 10.05	50	63.3 ± 3.03	76.48 ±14.51
25	41.69 ± 3.425	100	54.1 ± 4.39	64.12 ± 9.38
50	31.68 ± 4.346	200	64.74 ± 7.87	42.73 ± 5.6
100	21.95 ± 0.8226	400	29.81 ± 0.72	10.31 ± 1.8
200	17.37 ± 0.9953	800	11.62 ± 9.45	-3.31 ± 0.083
400	3.828 ± 0.7639	1600	0.33 ± 0.81	-3.68 ± 0.094
800	-0.9318 ± 0.2126	3200	-0.14 ± 2.65	-3.89 ± 0.053

± the standard deviation (SD) from three separate tests.

Table S2. Different amounts of COL-3), DOX, and MIN affect the health of KG-1a cells. The data are shown as the mean ± the SD from three separate tests.

The used COL-3 dose µg/ml	Percentage of viable cells after COL-3 treatment (Mean ±SD)	The used DOX and MIN dose µg/ml	Percentage of viable cells after DOX treatment (Mean ±SD)	Percentage of viable cells after MIN treatment (Mean ±SD)
0	100.3 ± 5.6	0	100.3 ± 5.5	100.3 ± 5.6
0.09	97.8 ± 2.5	0.7	101.2 ± 4.4	95.3 ± 5.7
0.18	108.4 ± 11.7	1.5	103.9 ± 8.6	96.4 ± 8.5
0.375	108.5 ± 17.3	3.12	115.3 ± 8.6	101.5 ± 4.5
0.75	104.2 ± 8.5	6.25	113 ± 7.2	104.2 ± 4.9
1.5	83.7 ± 7.1	12.5	83.1 ± 7	89.6 ± 7.4
3	73.2 ± 8.9	25	75 ± 4.1	74.2 ± 9.4
6	57.7 ± 1.9	50	62 ± 3.7	68.2 ± 3.2
12.5	39.8 ± 2.3	100	53 ± 5.9	59.2 ± 3.4
25	24.7 ± 1.8	200	35.6 ± 2.5	47.1 ± 3.7
50	15.2±1.7	400	10.2 ± 0.7	5.7 ± 0.9
100	3.9 ±1.1	800	-0.7 ± 0.6	-1.3 ± 0.05
200	-0.142 ± 1.3	1600	0.9 ± 0.3	-1.2 ± 0.3

Table S3. Different amounts of COL-3, DOX, and MIN affect the health of Jurkat cells. The data are shown as the mean ± the SD from three separate tests.

The used COL-3 dose µg/ml	The Percentage of viable cells after COL-3 treatment (Mean ±SD)	The used DOX and MIN dose µg/ml	The Percentage of viable cells after DOX treatment (Mean ±SD)	The Percentage of viable cells after MIN treatment (Mean ±SD)
0	100 ± 6.8	0	100 ± 6.8	100 ± 6.8
0.09	103.2 ± 6.0	0.7	112.6 ± 9.2	111.9 ± 4.8
0.18	102.4 ± 6.8	1.5	119.9 ± 7.4	110.3 ±9.5
0.375	100.9 ± 3.1	3.12	112.6 ± 10.6	104.8 ± 3.3
0.75	86.6 ± 0.8	6.25	80.4 ± 10.2	79.3 ± 3.5
1.5	71.8 ± 3.6	12.5	24.5 ± 1.8	41.5 ± 1.2
3	58.3 ± 0.4	25	3.7 ± 2.3	12.2 ± 1.4
6	36.1 ± 1.9	50	1.3 ± 1.6	5.0 ± 0.5
12.5	13.3 ± 1	100	0.4 ± 0.8	1.3 ± 0.1
25	6.1 ± 0.2	200	0.04 ± 0.2	2.1 ± 0.4
50	2.5 ± 0.4			
100	0.01 ± 0.1			

Table S4. Effect of COL-3, DOX, and MIN on the viability and apoptosis of K562 cells. The table shows the percentage of viable cells, annexin V-positive cells, 7-AAD-positive cells, and double-positive cells (annexin V and 7-AAD) after treatment. a: Statistically significant difference compared to 4-hour control cells (P < 0.05). b: Statistically significant difference compared to 24-hour control cells (P < 0.05).

	Cell line Cells	viable cells Mean ± SD	annexin +ve cells Mean ± SD	7-AAD +ve cells Mean ± SD	Double +ve cells Mean ± SD
COL-3	4hrs control	75.7 ± 0.5	10.6 ± 0.7	0.9 ± 0.1	11.7 ± 0.4
	24hrs control	46.1 ± 1.3	23 ± 1.1	0.5 ± 0.1	28.4 ± 0.3
	4hrs 12.5 µg/ml	71.1 ± 1.3[a]	16.4 ± 0.4[a]	3.7 ± 0.8[a]	7.6 ± 0.3
	24hrs 12.5 µg/ml	28.4 ± 1.8[b]	29.6 ± 0.7[b]	2.9 ± 0.4[b]	33.7 ± 3.1
	4hrs 25µg/ml	71.1 ± 1.3[a]	16.4 ± 0.4[a]	3.7 ± 0.8[a]	10.8 ±5.2
	24hrs 25µg/ml	15.4 ± 0.2[b]	32.1 ± 0.04[b]	2.1 ± 0.1[b]	33.1 ±0.8
DOX	4hrs control	75.7 ± 0.5	10.6 ± 0.7	0.9 ± 0.04	11.7 ± 0.5
	24hrs control	46.1 ± 1.3	23.1 ± 1.1	0.5 ± 0.1	28.4 ± 0.4
	4hrs 100 µg/ml	78.8 ± 0.2[a]	12.03 ± 0.2	0.1 ± 0.01[a]	8.3 ± 0.1a
	24hrs 100 µg/ml	46 ± 1.5	23.8 ± 1.3	0.2 ±0.1b	27.3 ± 1,2[b]
	4hrs 200 µg/ml	78.8 ± 0.2[a]	12.1 ± 0.2	0.1 ± 0.01[a]	8.2 ± 0.3
	24hrs 200 µg/ml	38.8 ± 0.6[b]	25.6 ± 0.9[b]	0.2 ± 0.1[b]	33.1 ±0.7[b]
MIN	4hrs control	75.7 ± 0.5	10.6 ± 0.7	0.9 ± 0.04	0.9 ± 0.04
	24hrs control	46.1 ± 1.3	23.1 ± 1.1	0.6 ± 0.1	0.6 ± 0.1
	4hrs 100 µg/ml	79.6 ± 0.2	9.8 ± 0.5	0.2 ± 0.1[a]	9.9 ± 0.4[a]
	24hrs 100 µg/ml	48.1 ± 0.4[b]	25.5 ± 0.7[b]	0.1 ± 0.01[b]	24.8 ± 0.7[b]
	4hrs 200 µg/ml	82.1 ± 0.1	8.9 ± 0.4	0.1 ± 0.01[a]	8.1 ± 0.2[a]
	24hrs 200 µg/ml	42.4 ± 0.9[b]	23.9 ± 0.5	0.2 ± 0.01[b]	31.8 ± 0.8[b]

Table S5. Effect of COL-3, DOX, and MIN on the viability and apoptosis of KG-1a cells. The table shows the percentage of viable cells, annexin V-positive cells, 7-AAD-positive cells, and double-positive cells (annexin V and 7-AAD) after treatment. a: Statistically significant difference compared to 4-hour control cells (P < 0.05). b: Statistically significant difference compared to 24-hour control cells (P < 0.05).

	Cell line type	viable cells Mean ± SD	annexin +ve cells Mean ± SD	7-AAD +ve cells Mean ± SD	Double +ve cells Mean ± SD
COL-3	4hrs control	99.4 ± 0.1	0.04 ± 0.3	0.9 ± 0.04	0.1 ± 0.03
	24hrs control	99.7 ± 0.01	0.02 ± 0.01	0.6 ± 0.1	0.2 ± 0.02
	4hrs 3µg/ml	94.9 ± 2.2[a]	4.3 ± 2.1[a]	0.4 ± 0.1[a]	0.3 ± 0.03[a]
	24hrs 3 µg/ml	88.3 ± 0.5[b]	4.7 ± 0.5[b]	0.5 ± 0.1	0.5 ± 0.1[b]
	4hrs 6µg/ml	93.7 ± 3.3[a]	5.6 ± 3.3[a]	0.4 ± 0.1[a]	0.3 ± 0.04[a]
	24hrs 6µg/ml	82.9 ± 0.1[b]	15.6 ± 1.5[b]	2.2 ± 0.1[b]	1.9 ± 0.1[b]
DOX	4hrs control	99.4 ± 0.1	0.04 ± 0.3	0.9 ± 0.04	0.1 ± 0.03
	24hrs control	99.7 ± 0.01	0.02 ± 0.01	0.6 ± 0.1	0.2 ± 0.02
	4hrs 50 µg/ml	98.4 ± 0.1[a]	1.2 ± 0.1[a]	0.3 ± 0.04[a]	0.01 ± 0.01[a]
	24hrs 50 µg/ml	81.4 ± 0.2[b]	14.5 ± 0.3[b]	0.3 ± 0.04[b]	3.6 ± 0.2[b]
	4hrs 100 µg/ml	97.7 ± 0.2[a]	1.8 ± 0.1[a]	0.2 ± 0.1[a]	0.2 ± 0.1[a]
	24hrs 100 µg/ml	76.1 ± 0.2[b]	17.2 ± 0.1[b]	1 ± 0.1[b]	5.4 ± 0.2[b]
MIN	4hrs control	99.4 ± 0.1	0.04 ± 0.3	0.9 ± 0.04	0.1 ± 0.03
	24hrs control	99.7 ± 0.01	0.02 ± 0.01	0.6 ± 0.1	0.2 ± 0.02
	4hrs 100 µg/ml	99.5 ± 0.1	0.05 ± 0.01	0.2 ± 0.1	0.2 ± 0.05
	24hrs 100 µg/ml	74 ± 0.7[b]	18 ± 0.4[b]	0.5 ± 0.03	7.2 ± 0.2[b]
	4hrs 200 µg/ml	99.1 ± 0.1	0.05 ± 0.02	0.4 ± 0.02	0.3 ± 0.1
	24hrs 200 µg/ml	52 ± 0.5[b]	23.1 ± 0.2[b]	0.6 ± 0.1	23.5 ± 0.4[b]

Table S6. Effect of COL-3, DOX, and MIN on the viability and apoptosis of Jurkat cells. The table shows the percentage of viable cells, annexin V-positive cells, 7-AAD-positive cells, and double-positive cells (annexin V and 7-AAD) after treatment. a: Statistically significant difference compared to 4-hour control cells (P < 0.05). b: Statistically significant difference compared to 24-hour control cells (P < 0.05).

	Cell line Cells	viable cells Mean ± SD	annexin +ve cells Mean ± SD	7-AAD +ve cells Mean ± SD	Double +ve cells Mean ± SD
COL-3	4hrs control	95.6 ± 0.1	0.6 ± 0.2	1.2 ± 0.4	2.5 ± 0.4
	24hrs control	83.7 ± 0.5	4.2 ± 1.2	0.9 ± 0.4	11 ± 0.7
	4hrs 1.5 µg/ml	79.6 ± 0.4[a]	0.8 ± 0.07	3.4 ± 0.2	15.9 ± 0.6[a]
	24hrs1.5 µg/ml	55.8 ± 1.4[b]	9.7 ± 0.3[b]	5 ± 0.5[b]	29 ± 0.9[b]
	4hrs 3µg/ml	66.5 ± 0.9[a]	0.7 ± 0.2	8.8 ± 0.6[a]	22.9 ± 0.7[a]
	24hrs 3µg/ml	52.6 ± 1.7[b]	9.9 ± 2[b]	17.9 ± 3.6[b]	19 ± 0.7[b]
DOX	4hrs control	95.6 ± 0.1	0.6 ± 0.2	1.2 ± 0.4	2.5 ± 0.4
	24hrs control	83.7 ± 0.5	4.2 ± 1.2	0.9 ± 0.1	11 ± 0.7
	4hrs 7 µg/ml	89 ± 0.2[a]	1.46 ± 0.01	1.3 ± 0.2	8.1 ± 0.1[a]
	24hrs 7 µg/ml	65.6 ± 1.8[b]	8.4 ± 0.12[b]	1.6 ± 1.1	24 ± 0.8[b]
	4hrs 10 µg/ml	85 ± 1[a]	1.513 ± 0.06	2.1 ± 0.4	11.2 ± 0.9[a]
	24hrs 10 µg/ml	59.4 ± 1.3[b]	10.4 ± 0.3[b]	1.1 ± 0.2	28.5 ± 1.1[b]
MIN	4hrs control	95.6 ± 0.2	0.6 ± 0.2	1.2 ± 0.4	2.5 ± 0.4
	24hrs control	83.7 ± 0.5	4.2 ± 1.2	0.8 ± 0.1	11 ± 0.7
	4hrs 10 µg/ml	68.3 ± 1[a]	6.3 ± 0.6[a]	4 ± 0.3[a]	20.9 ± 0.7[a]
	24hrs 10 µg/ml	49.4 ± 1.3[b]	28.8 ± 0.8[b]	0.7 ± 0.1	20.6 ± 0.5[b]
	4hrs 12 µg/ml	66.2 ± 0.9[a]	6.5 ± 1[a]	4 ± 0.6[a]	22.8 ± 0.9[a]
	24hrs 12 µg/ml	48.1 ± 0.7[b]	28.6 ± 0.4[b]	0.8 ± 0.2	22 ± 0.1[b]

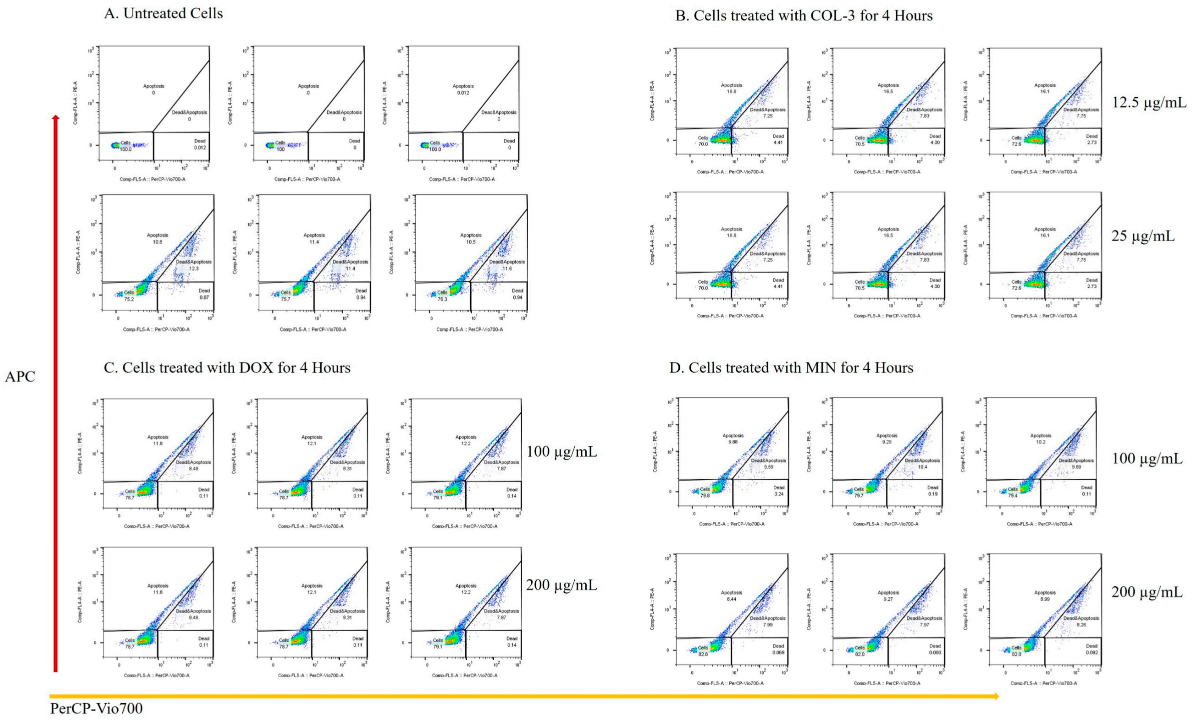

Supplementary Figure S1. Effect of COL-3, DOX, and MIN on apoptosis in K562 cells assessed by flow cytometry. Apoptosis was evaluated using Annexin V and 7-AAD staining followed by flow cytometric analysis. (A) Untreated control cells: upper panel shows unstained cells; lower panel displays the distribution of Annexin V–positive (early apoptotic), 7-AAD–positive (late apoptotic/necrotic), and double-stained (late apoptotic/necrotic) cells. (B) Cells treated with COL-3 at 12.5 µg/mL (upper panel) and 25 µg/mL (lower panel) for 4 hours. (C) Cells treated with DOX at 100 µg/mL (upper panel) and 200 µg/mL (lower panel) for 4 hours. (D) Cells treated with MIN at 100 µg/mL (upper panel) and 200 µg/mL (lower panel) for 4 hours. The percentage of cells in each quadrant reflects the extent of early and late apoptosis or necrosis under each treatment condition.

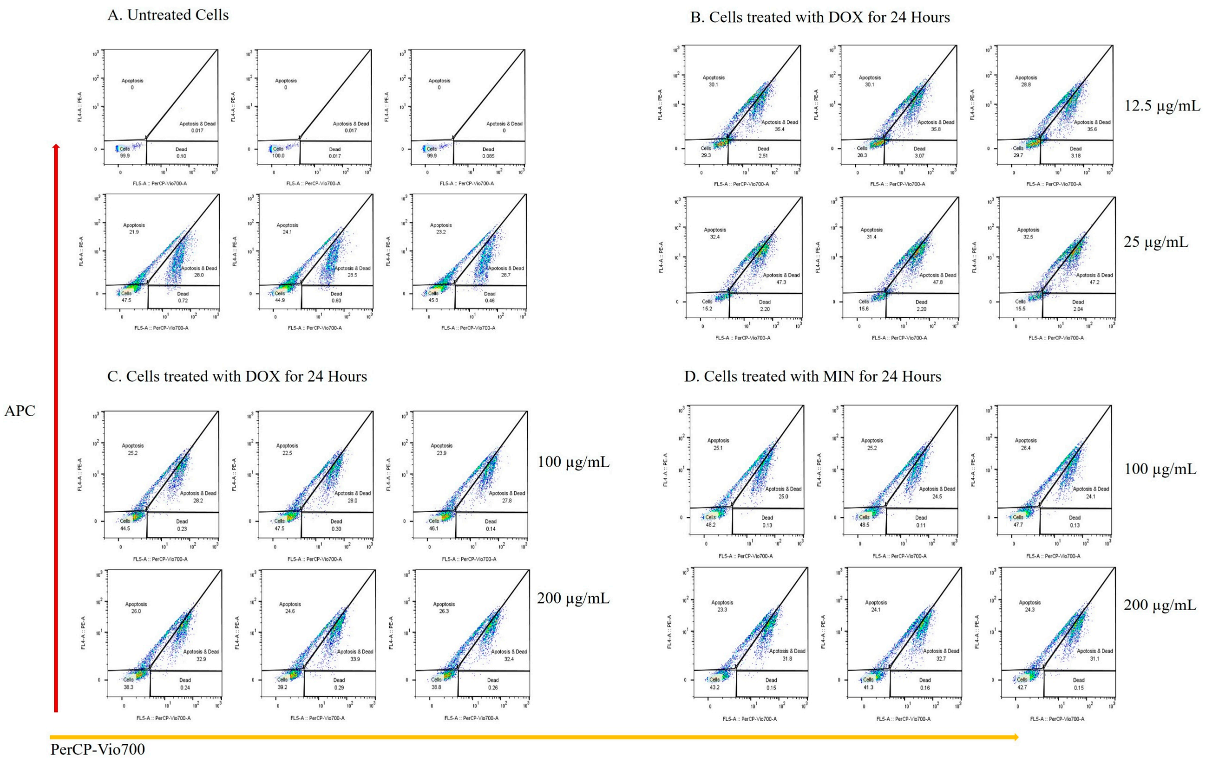

Supplementary Figure S2. Effect of COL-3, DOX, and MIN on apoptosis in K562 cells assessed by flow cytometry. Apoptosis was evaluated using Annexin V and 7-AAD staining followed by flow cytometric analysis. (A) Untreated control cells: upper panel shows unstained cells; lower panel displays the distribution of Annexin V–positive (early apoptotic), 7-AAD–positive (late apoptotic/necrotic), and double-stained (late apoptotic/necrotic) cells. (B) Cells treated with COL-3 at 12.5 µg/mL (upper panel) and 25 µg/mL (lower panel) for 24 hours. (C) Cells treated with DOX at 100 µg/mL (upper panel) and 200 µg/mL (lower panel) for 24 hours. (D) Cells treated with MIN at 100 µg/mL (upper panel) and 200 µg/mL (lower panel) for 24 hours. The percentage of cells in each quadrant reflects the extent of early and late apoptosis or necrosis under each treatment condition.

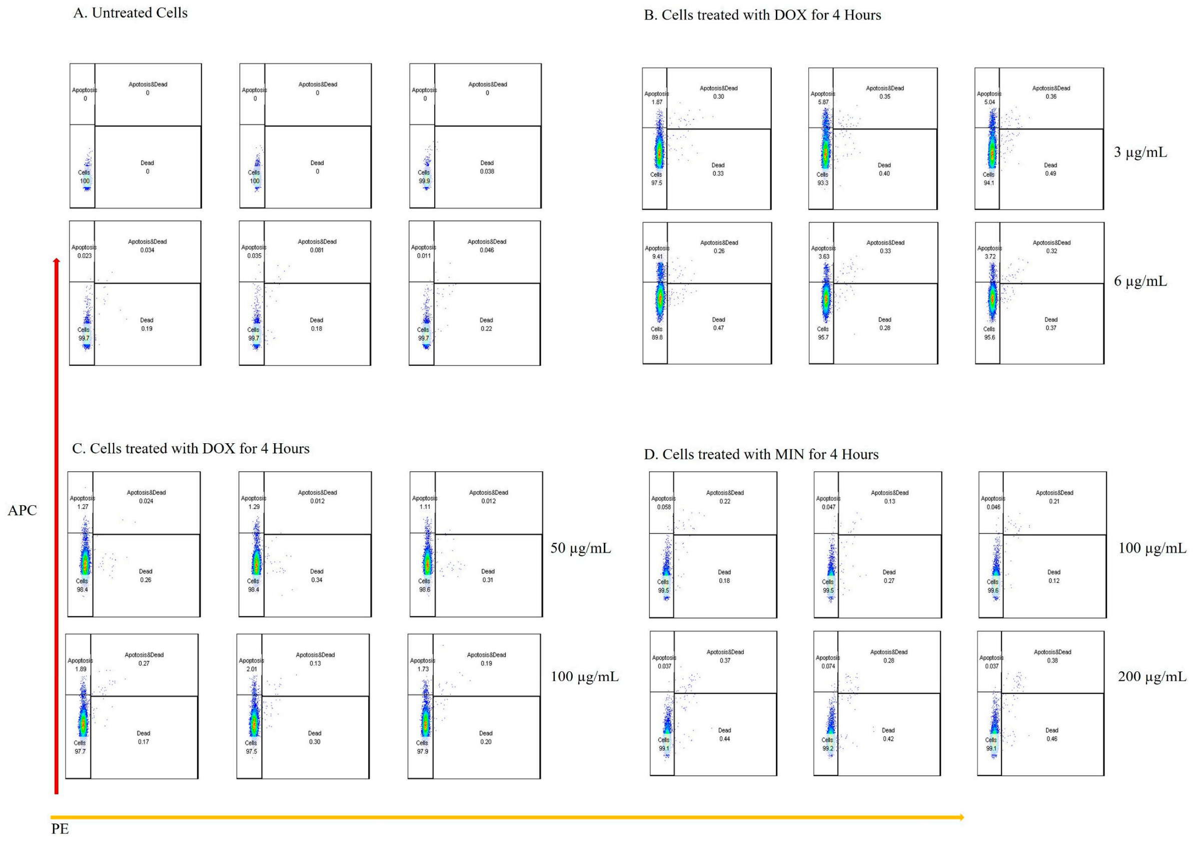

Supplementary Figure S3. Effect of COL-3, DOX, and MIN on apoptosis in KG-1a cells assessed by flow cytometry. Apoptosis was evaluated using Annexin V and 7-AAD staining followed by flow cytometric analysis. (A) Untreated control cells: upper panel shows unstained cells; lower panel displays the distribution of Annexin V–positive (early apoptotic), 7-AAD–positive (late apoptotic/necrotic), and double-stained (late apoptotic/necrotic) cells. (B) Cells treated with COL-3 at 3 µg/mL (upper panel) and 6 µg/mL (lower panel) for 4 hours. (C) Cells treated with DOX at 50 µg/mL (upper panel) and 100 µg/mL (lower panel) for 4 hours. (D) Cells treated with MIN at 100 µg/mL (upper panel) and 200 µg/mL (lower panel) for 4 hours. The percentage of cells in each quadrant reflects the extent of early and late apoptosis or necrosis under each treatment condition.

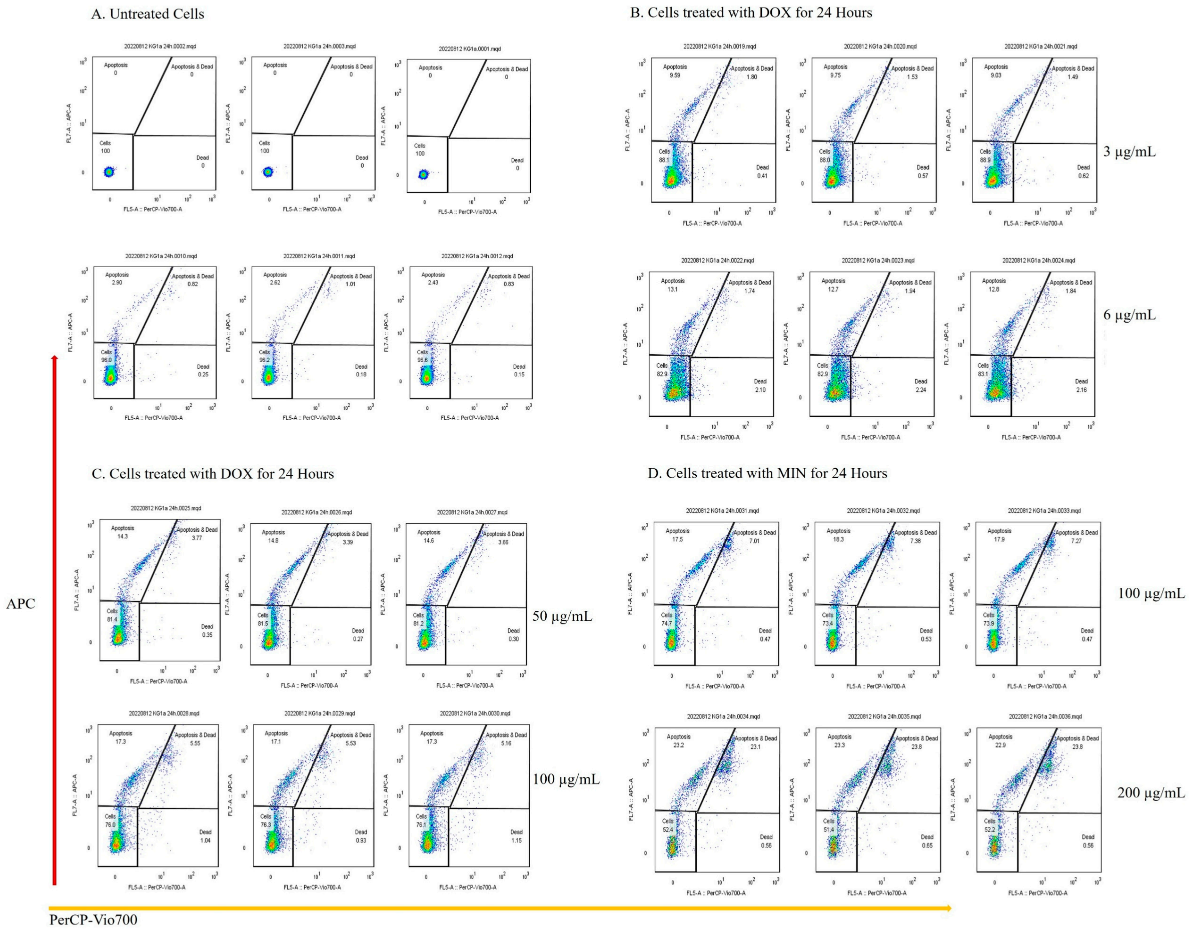

Supplementary Figure S4. Effect of COL-3, DOX, and MIN on apoptosis in KG-1a cells assessed by flow cytometry. Apoptosis was evaluated using Annexin V and 7-AAD staining followed by flow cytometric analysis. (A) Untreated control cells: upper panel shows unstained cells; lower panel displays the distribution of Annexin V–positive (early apoptotic), 7-AAD–positive (late apoptotic/necrotic), and double-stained (late apoptotic/necrotic) cells. (B) Cells treated with COL-3 at 3 µg/mL (upper panel) and 6 µg/mL (lower panel) for 24 hours. (C) Cells treated with DOX at 50 µg/mL (upper panel) and 100 µg/mL (lower panel) for 24 hours. (D) Cells treated with MIN at 100 µg/mL (upper panel) and 200 µg/mL (lower panel) for 24 hours. The percentage of cells in each quadrant reflects the extent of early and late apoptosis or necrosis under each treatment condition.

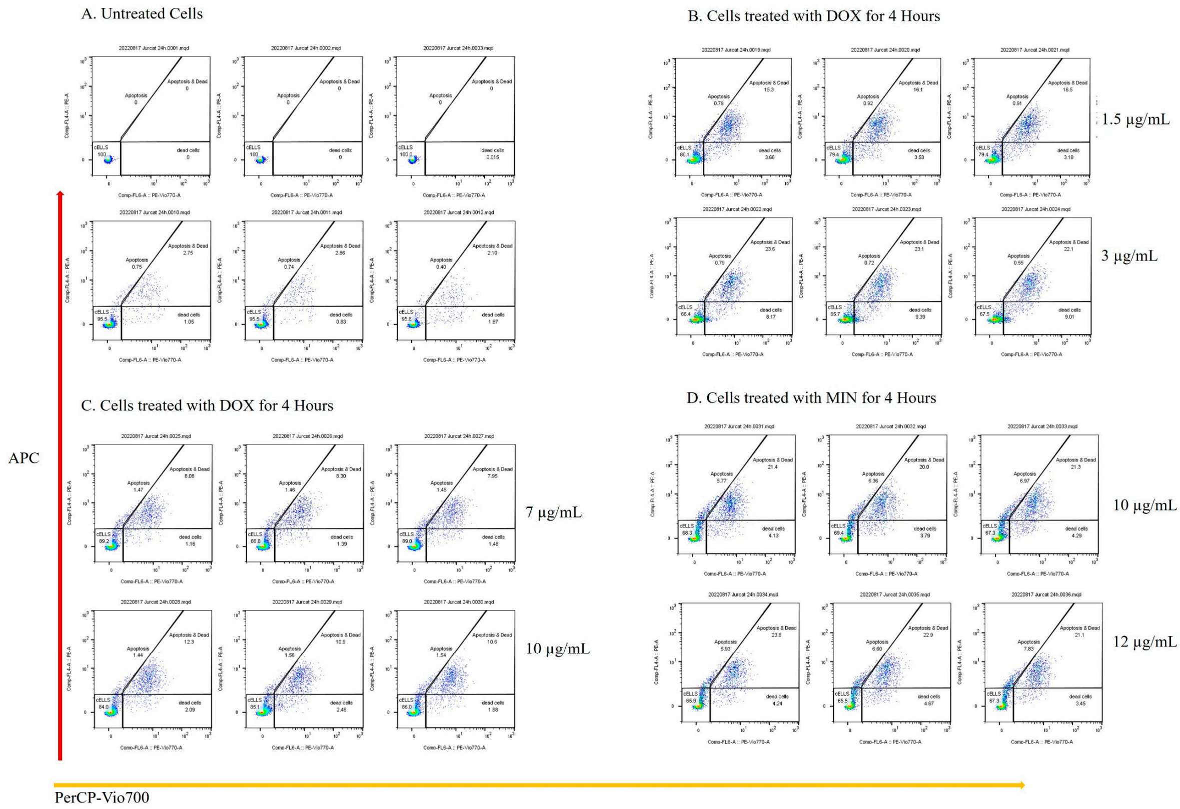

Supplementary Figure S5. Effect of COL-3, DOX, and MIN on apoptosis in Jurkat cells assessed by flow cytometry. Apoptosis was evaluated using Annexin V and 7-AAD staining followed by flow cytometric analysis. (A) Untreated control cells: upper panel shows unstained cells; lower panel displays the distribution of Annexin V–positive (early apoptotic), 7-AAD–positive (late apoptotic/necrotic), and double-stained (late apoptotic/necrotic) cells. (B) Cells treated with COL-3 at 1.5 µg/mL (upper panel) and 3 µg/mL (lower panel) for 4 hours. (C) Cells treated with DOX at 7 µg/mL (upper panel) and 10 µg/mL (lower panel) for 4 hours. (D) Cells treated with MIN at 10 µg/mL (upper panel) and 12 µg/mL (lower panel) for 4 hours. The percentage of cells in each quadrant reflects the extent of early and late apoptosis or necrosis under each treatment condition.

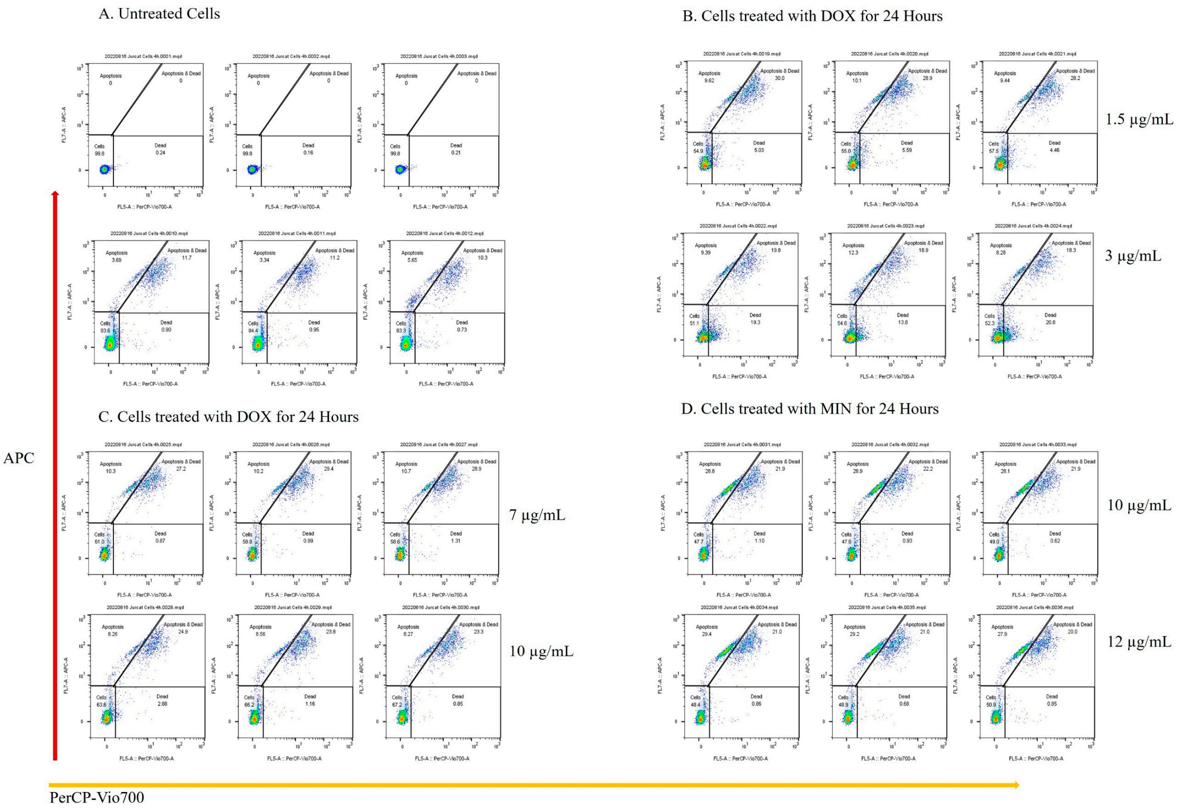

Supplementary Figure S6. Effect of COL-3, DOX, and MIN on apoptosis in Jurkat cells assessed by flow cytometry. Apoptosis was evaluated using Annexin V and 7-AAD staining followed by flow cytometric analysis. (A) Untreated control cells: upper panel shows unstained cells; lower panel displays the distribution of Annexin V–positive (early apoptotic), 7-AAD–positive (late apoptotic/necrotic), and double-stained (late apoptotic/necrotic) cells. (B) Cells treated with COL-3 at 1.5 μg/mL (upper panel) and 3 μg/mL (lower panel) for 24 hours. (C) Cells treated with DOX at 7 μg/mL (upper panel) and 10 μg/mL (lower panel) for 24 hours. (D) Cells treated with MIN at 10 μg/mL (upper panel) and 12 μg/mL (lower panel) for 24 hours. The percentage of cells in each quadrant reflects the extent of early and late apoptosis or necrosis under each treatment condition.

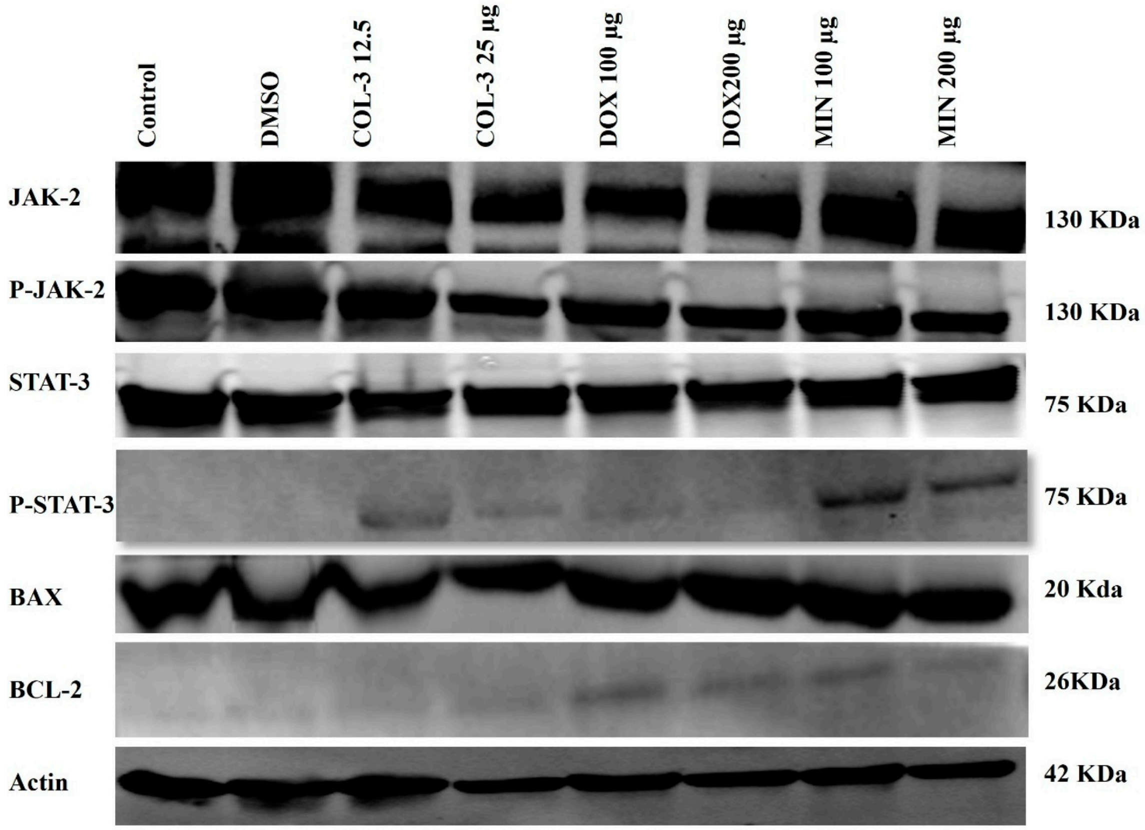

Supplementary Figure S7. Tetracycline Analogues modulate the JAK2/STAT3 signalling pathway in treated K562 cells. Western blot analysis was conducted to evaluate the expression levels of total and phosphorylated JAK2 and STAT3 (JAK2, P-JAK2, STAT3, P-STAT3), as well as the pro-apoptotic marker BAX and the anti-apoptotic marker BCL-2. Cells were treated for 24 hours with COL-3 at final concentrations of 12.5 and 25 µg/mL, DOX at 100 and 200 µg/mL, or MIN at 100 and 200 µg/mL. DMSO (0.2%) was used as a vehicle control for COL-3-treated cells, while untreated cells cultured in complete medium (CO) served as controls for DOX and MINO treatments. β-actin was used as a loading control. Protein expression levels are presented as fold changes relative to the respective control group for each treatment condition.

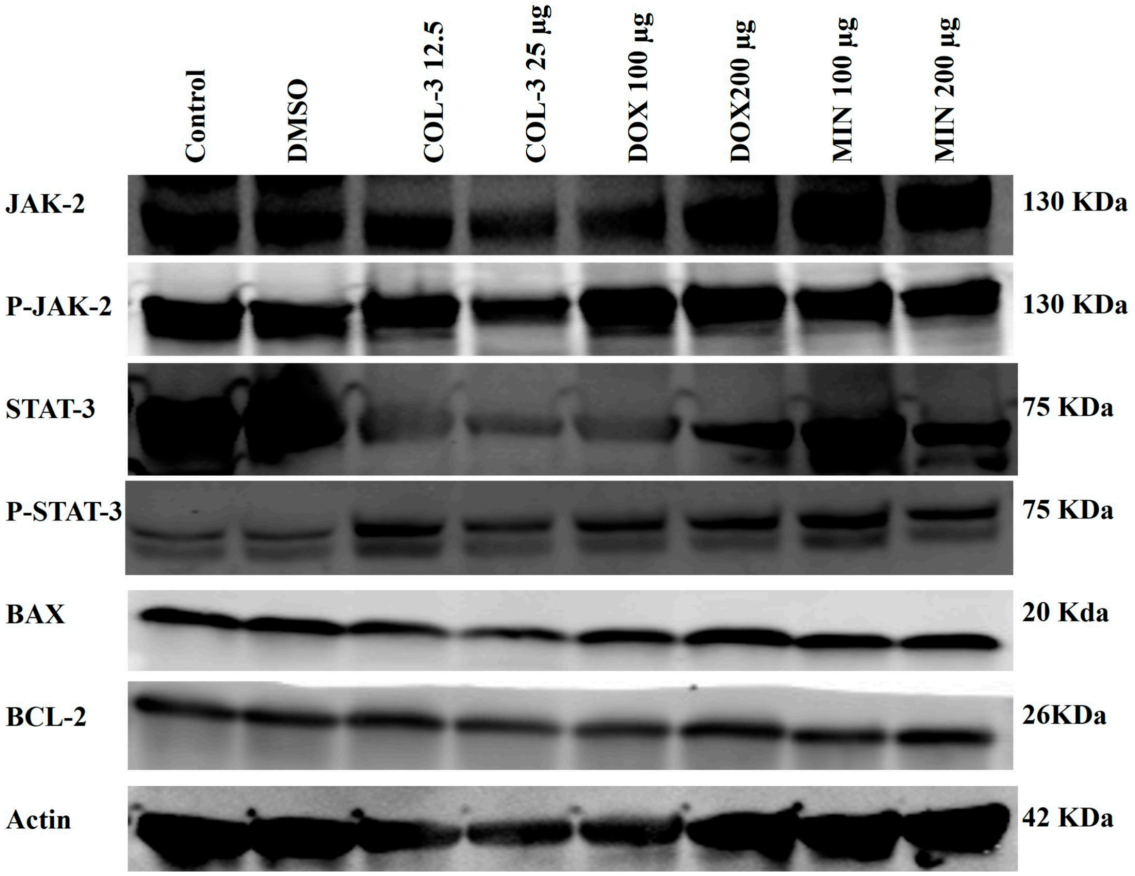

Supplementary Figure S8. Tetracycline Analogues modulate the JAK2/STAT3 signaling pathway in treated KG-1a cells. Western blot analysis was conducted to evaluate the expression levels of total and phosphorylated JAK2 and STAT3 (JAK2, P-JAK2, STAT3, P-STAT3), as well as the pro-apoptotic marker BAX and the anti-apoptotic marker BCL-2. Cells were treated for 24 hours with COL-3 at final concentrations of 12.5 and 25 µg/mL, DOX at 100 and 200 µg/mL, or MIN at 100 and 200 µg/mL. DMSO (0.2%) was used as a vehicle control for COL-3-treated cells, while untreated cells cultured in complete medium (CO) served as controls for DOX and MINO treatments. β-actin was used as a loading control. Protein expression levels are presented as fold changes relative to the respective control group for each treatment condition.

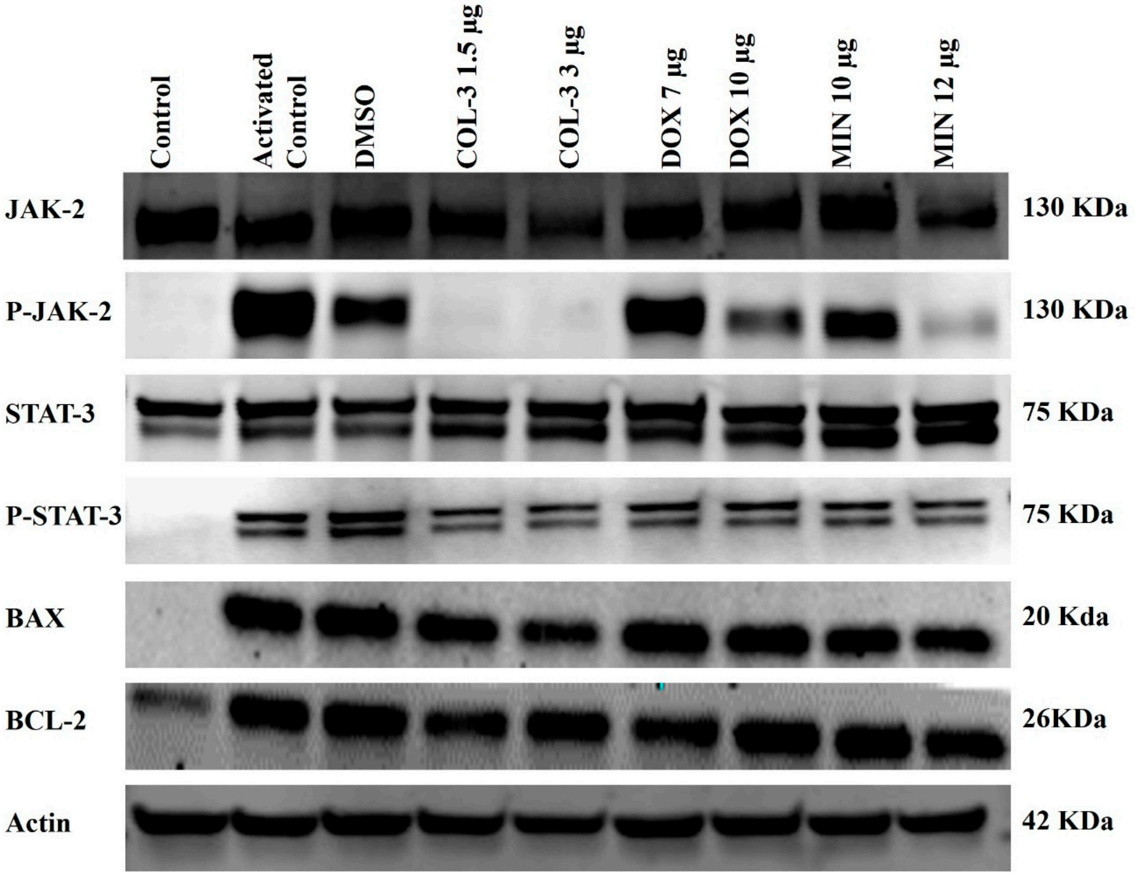

Supplementary Figure S9. Tetracycline Analogues modulate the JAK2/STAT3 signaling pathway in treated Jurkat cells. Western blot analysis was conducted to evaluate the expression levels of total and phosphorylated JAK2 and STAT3 (JAK2, P-JAK2, STAT3, P-STAT3), as well as the pro-apoptotic marker BAX and the anti-apoptotic marker BCL-2. Cells were treated for 24 hours with COL-3 at final concentrations of 1.5 and 3 µg/mL, DOX at 7 and 10 µg/mL, or MIN at 10 and 12 µg/mL. DMSO (0.2%) was used as vehicle control for COL-3-treated cells, while untreated cells cultured in complete medium (CO^+) served as controls for DOX and MINO treatments. β-actin was used as a loading control. Protein expression levels are presented as fold changes relative to the respective control group for each treatment condition.
